# Technology-Enabled Cognitive Strategy Intervention for Secondary Stroke Prevention: A Feasibility Study

**DOI:** 10.3390/bioengineering12070778

**Published:** 2025-07-18

**Authors:** Timothy Dionne, Jessica D. Richardson, Davin Quinn, Karen Luo, Suzanne Perea Burns

**Affiliations:** Center for Brain Recovery and Repair, University of New Mexico School of Medicine, Albuquerque, NM 87131, USA; tdionne@salud.unm.edu (T.D.); jdrichardson@unm.edu (J.D.R.); dquinn@salud.unm.edu (D.Q.); kaluo@salud.unm.edu (K.L.)

**Keywords:** stroke, stroke rehabilitation, cognition, vascular risk factors, heart disease, dementia

## Abstract

Background: People with post-stroke cognitive impairment (PSCI) are at increased risk of recurrent stroke, dementia, and accelerated cognitive decline. Objective: To examine the feasibility, safety, acceptability, and suitability of a virtually-delivered vascular risk reduction intervention that integrates tailored cognitive strategy training for people with executive function (EF) impairments post-stroke. Methods: This case series included eight participants who completed up to ten virtual sessions focused on vascular risk reduction and metacognitive strategy training. Sessions averaged 40 min over a 4–5-week period. Results: The intervention was found to be feasible, safe, and acceptable. The recruitment rate was 66.7%, and the retention rate was 87.5% (7 of 8 completed the training). No serious adverse events were reported. Most participants demonstrated improvements on the Canadian Occupational Performance Measure (COPM), with mean performance and satisfaction change scores of 1.22 ± 0.87 and 1.18 ± 0.83, respectively. Conclusions: This technology-enabled intervention was feasible and acceptable for individuals with post-stroke EF impairments. Virtual delivery was a key factor in its accessibility and success. The results are promising for improving self-management of vascular risk factors, warranting further study in larger trials.

## 1. Introduction

Each year, nearly 795,000 Americans experience a stroke, with approximately 185,000 cases being a recurrent event [1]. Stroke is the fifth leading cause of death and a major leading cause of disability in the United States (US) [1]. Projections predict a 20.5% increase in the prevalence of persons with stroke from 2012 to 2030 [2]. Survivors of stroke report persistent unmet long-term needs after discharge to the community [3,4,5]. Many people with stroke are discharged home without services and must learn to manage their own health and recovery [6]. This is particularly challenging for persons with post-stroke cognitive impairment (PSCI) who have impaired cognitive processes that negatively influence their ability to plan, make decisions, and engage in daily tasks like preparing a meal, crossing a street, managing medications, and resuming life roles in the community [6,7]. This is noteworthy as rehabilitation efforts often fall short as more than 60% of survivors discontinue engagement in meaningful life roles [8].

PSCI is highly disabling. People with PSCI encounter major challenges when engaging in daily tasks and reintegrating to the community [4,7,9,10]. While as much as 80% of the population of people living with stroke have some degree of cognitive impairment [11,12,13,14], it is notable that cognitive impairments, like executive function (EF) deficits, often do not emerge until after resuming life in the community where the structure of life in the hospital is absent [15,16]. EF is a collection of cognitive processes that include orienting towards the future (i.e., planning), demonstrating self-control (i.e., behavioral inhibition), problem solving, adapting to environmental changes, and facilitating goal-directed behaviors, all of which are essential skills for managing health [15,17,18]. This is highly concerning as people with EF deficits may not be receiving treatments due to lack of identification of deficits. Unfortunately, the risk of cognitive decline and dementia is high with stroke. Approximately 10% develop dementia prior to the stroke, 10% shortly after the stroke, and greater than 1/3 of the patients with recurrent stroke are classified as having dementia [19,20]. People with cognitive impairments like EF deficits are at an increased risk for progression to post-stroke dementia and are also at significant risk of cognitive decline with subsequent strokes [20].

While risk reduction education is a key element of stroke prevention, it often relies on didactic education and assumes intact executive functioning. However, individuals with post-stroke EF impairments may struggle to translate knowledge into action due to difficulties with skills like planning, self-monitoring, and problem solving. Cognitive strategies for improving Health outcomes And Managing risk Post-Stroke (CHAMPS) was designed to address this problem by combining vascular risk reduction education with cognitive strategy training. This novel integration may enhance the ability to adopt and sustain health behaviors which are critical to reducing secondary stroke risk among persons with EF impairments.

The primary goal of cognitive rehabilitation is to ameliorate impairments to enhance safety, independence, participation, and quality of life [21,22,23]. Cognitive rehabilitation supports metacognitive self-regulation strategy training (e.g., “goal setting, planning, initiation, execution, self-monitoring, and error management”) for deficits in EF [23]. A necessary consideration when implementing interventions in persons with stroke is that the associated impairment can influence acquisition (learning purpose and procedures of strategy), application (practicing applying strategies to simple tasks), and adaptation (applying strategies to everyday tasks outside of treatment environment) of an intervention, hindering self-management of positive health behaviors [21]. Metacognitive training, such as the training in the CHAMPS intervention, facilitates neuroplastic changes in neuronal pathways through repeated engagement in meaningful and salient tasks that are necessary for skill acquisition and goal training [24]. In fact, there is evidence for positive increases in neuronal activity in those with cancer-related cognitive impairment [25].

Vascular risk reduction is effective for optimizing brain health and preventing stroke. Brain health is defined as the “optimal capacity to function adaptively in the environment” and both stroke and cerebrovascular disease are becoming more widely recognized as precursors to cognitive decline and dementia (i.e., “absence of brain health”) [1,26]. The American Heart Association (AHA)’s cardiovascular health metrics (Life’s Essential 8; LE8) includes information on modifiable risk factors that can contribute to ideal vascular health and have been associated with a lower incidence of cognitive impairment [27,28,29].

Stroke doubles the risk of cognitive decline and dementia, which can either occur immediately following the stroke or may involve an accelerated decline over time [19,30]. Although cognitive decline and/or dementia can occur for a number of reasons after stroke (e.g., infarcts in strategic areas and multiple strokes), several studies have emphasized the influence of stroke and related vascular risk factors on cognitive decline and dementia [31]. White matter hyperintensities (WMHs) of vascular origin are commonly identified in MRIs of even healthy adults. Unfortunately, WMHs increase the risk of functional decline, stroke, cognitive decline, and even death [31]. Controlling vascular risk is important for recurrent stroke prevention and may also delay or prevent cognitive decline or dementia. For instance, the Finnish Geriatric Intervention Study to Prevent Cognitive Impairment and Disability (FINGER) trial addressed dementia risk factors through cardiovascular risk management and cognitive training to protect brain health in older adults at risk for dementia and found that the multi-domain intervention had beneficial effects on primary cognitive outcomes despite the presence of cardiovascular comorbidities (e.g., stroke and myocardial infarction) [32]. Unfortunately, healthcare providers do not typically consider how disability status impacts how education is transferred and applied to everyday life [33] and it is critical to consider ways to effectively manage vascular disease risk factors to reduce the cardiovascular/dementia risk profile after stroke [32].

Advances in the acute management of stroke have led to more people surviving a stroke; however, there is a high prevalence of people living with the cognitive effects of stroke, which can be particularly challenging to manage and can lead to further health and cognitive decline [9]. CHAMPS may be particularly beneficial among adults with post-stroke EF impairments who are frequently discharged home but often have poor functional and long-term cognitive outcomes. The proposed intervention may prove to be a pragmatic and powerful strategy that can be scaled to providers in various institutions and communities seeking to improve stroke recovery outcomes. Thus, this study aimed to evaluate the feasibility, acceptability, and suitability of the novel CHAMPS intervention in persons with post-stroke EF impairments.

## 2. Methods

This study employed a case series design. The intervention was delivered virtually, and the outcome measurements were conducted in person.

### 2.1. Study Population

Participants were recruited between February and May 2024 through three pathways: (1) direct contact through a UNM acquired brain injury clinical registry, (2) direct contact through a university health system data warehouse utilizing medical records, and (3) community-based recruitment. The inclusion criteria were as follows: self-reported and confirmed stroke from medical records and registry information, and EF impairments from telephone EXIT interviews [34,35]. Participants were excluded if they were younger than 18 years of age; could not speak, read, and understand English; misused alcohol or drugs in the past 3 months (AUDIT [36], DAST [37]); had major/severe depression or anxiety (PHQ-9 [38], GAD-7 [39]); had pre-existing dementia (self-reported); or had severe aphasia (National Institute of Health Stroke Scale; NIHSS [40,41]). In feasibility trials, a priori analysis for sample size is not necessary as the focus is not on efficacy, but to assess the potential for successful execution in a scaled trial. While there is no universally accepted number of participants for a feasibility study [42], many feasibility studies have less than 10 participants. This study was approved by the UNM Institutional Review Board and all participants provided written informed consent prior to participation.

### 2.2. Intervention

The CHAMPS intervention consisted of up to 10 bi-weekly 30-to-60 min virtual treatment sessions. The sessions were administered by master’s-level occupational therapy (OT) student research personnel trained by SB and TD. The custom-tailored intervention targeted each participant’s self-identified vascular risk reduction and functional daily task goals while following the CHAMPS protocol which focused on AHA LE8 coaching on relevant topics including eating better, being more active, quitting tobacco, getting healthy sleep, managing weight, controlling cholesterol, managing blood sugar, and managing blood pressure [43,44]. The participants were provided with binders with all the educational content and strategy training information. It is important to note that while the binders included recommendations and instructions, the interventionists tailored the materials and coaching to each participant’s unique needs (e.g., literacy). The protocol was flexible and the participants engaged in 8–10 sessions in total. Participants were not required to cover content that was irrelevant to their personal health behaviors (e.g., non-smokers did not complete the section on smoking cessation). The 30–60 min total time remained flexible as diverse participants had differing abilities and needs. For instance, the session duration was variable due to differences in communication needs (e.g., aphasia), comfort with technology, complexity of personalized goals, and the amount of support needed for learning the metacognitive strategy. The cognitive strategy training was based on elements of the Cognitive Orientation to daily Occupational Performance (CO-OP) Approach^TM^ (i.e., goal setting, guided discovery, strategy development). Individual self-identified goals were developed using the Canadian Occupational Performance Measure (COPM) [45]. Each participant was required to include at least one goal related to the AHA LE8 content. The cognitive strategy training included guided discovery related to experiences relevant to the identified goals and training on the use of metacognitive strategies (thinking about thinking) and emphasizing potential problem-solving challenges using self-generated strategy development. At the end of each session, the participants were encouraged to engage in homework where they self-identified a weekly goal and integrated the learned strategy to support their success. Each session included a re-cap of the previous session and check in with homework and progress on weekly and long-term goals. Support persons like caregivers and family members were encouraged to join the sessions as possible.

To facilitate remote delivery of the cognitive strategy training intervention, the study employed Zoom Video Communications (Version 5.0.2), a HIPAA-compliant telehealth platform that has been widely adopted in clinical and rehabilitation settings. Zoom enabled synchronous, face-to-face engagement between the participants and interventionists, supporting the visual and verbal interaction essential for strategy modeling, cognitive rehearsal, and collaborative goal setting. Vascular risk reduction delivered via telehealth is not novel [43]; however, many of existing programs neglect to consider the implications of EF impairments for successful application of risk reduction strategies. The CHAMPS intervention stands in contrast to standard health coaching, which largely relies on didactic education and assumes preserved EF. CHAMPS is comprised of strategy training to support impairments in areas such as working memory, inhibitory control, and cognitive flexibility.

Usability considerations were central to the intervention design. The participants underwent structured onboarding to ensure familiarity with the Zoom interface, and the sessions followed a predictable format with consistent visual and auditory cues to reduce the cognitive load. The use of scheduled calendar invites and automated reminders functioned as compensatory aids to support EF. Accessibility was also tailored to each person’s needs within the binder (e.g., brightly colored sticky notes, attached pen). These design elements reflect the best practices in technology-assisted cognitive rehabilitation, aligning with the broader principles of universal design and digital health equity. By embedding cognitive prosthetic strategies within the telehealth medium itself, the intervention not only delivered the content remotely, but it also modeled how technology can function as an active therapeutic agent in supporting independence and strategy generalization

Although we anticipated that the goals would remain consistent throughout the program, the participants continuously identified new challenges with their vascular risk reduction, which led to setting new goals in the final sessions that were focused on the newly identified problems (e.g., medication management and reducing dietary sugar intake). Refer to Table 1 for intervention details and elements.

### 2.3. Assessment

Baseline and post-intervention testing were conducted in person UNM. To accommodate participant difficulties with community mobility, testing visits were modified to include in-home visits and virtual video-conference visits for post-intervention testing when requested.

Feasibility Assessment. Feasibility was the primary outcome of this study. Feasibility was evaluated using recruitment, adherence, and dropout rates. The methods for identifying, screening, and enrolling participants were evaluated using detailed study records. Acceptability was evaluated through adherence and retention rates using detailed study records and documentation. After ensuring that all interventionists received standardized training, fidelity was assessed through detailed checklists and study logs, descriptions of the intervention components in a REDCap (Version 14.0.1) documentation system, and through direct observation where the PI attended 5% of the sessions. Suitability was evaluated using the Suitability Assessment of Materials (SAM) [46].

Baseline characteristics. Sociodemographic data were collected using validated questions from the Behavioral Risk Factor Surveillance System (BRFSS) [47]. The participants were characterized with a series of EF assessments which included the following.

Select EF tests from the National Institute of Health (NIH) Toolbox Cognition Battery, a computer-administered tool to test cognition across studies, were used. Their reliability and validity in people with neurologic disorders including stroke have been established [48,49,50,51]. The following domains were assessed: cognitive flexibility and attention (Dimensional Change Card Sort Test), inhibitory control and attention (Flanker Task), working memory (List Sorting), and processing speed (Pattern Comparison).

The Oxford Multiple Errands Test (OxMET) is a mobile app-based assessment that screens for the effects of executive dysfunction through a simulated shopping scenario. The standardized tool was normed and validated in community-dwelling adults and those with stroke [52,53,54,55].

The Menu Task Assessment is a brief screening tool that evaluates functional cognitive impairments and is both reliable and valid in adults for detecting cognitive impairments [56,57,58].

Risk Factors. Vascular risk factors were evaluated using the American Heart Association’s guidelines for each LE8 component metric including physical activity, Body Mass Index (BMI), blood pressure, glucose/diabetes, cholesterol level, nicotine exposure, sleep health, and diet [59,60]. Scores were calculated using a trichotomous technique where the score for each LE8 element was categorized as poor (0), intermediate (1), or ideal (2) [61]. This approach was used with the AHA Life’s Simple 7 (LS7) and was modified to include sleep, which was classified as poor (≤4 h per night), intermediate (5–7 h per night), and ideal (≥7 h per night). Data were collected trichotomously, aligning with the LS7 methodology [62]. The total scores ranged from 0 to 16, with lower scores indicating a greater risk.

Dementia risk factor scores were calculated using the Cardiovascular Risk Factors, Aging, and Dementia (CAIDE) [63] modified CAIDE (mCAIDE) [64] risk score using a 0–14 scale where 14 indicates the highest risk. The scores are based on the following factors: age, education, systolic blood pressure, BMI, self-reported high cholesterol, and level of physical activity. The identified cut-off for risk is ≥7 [64].

Self-Efficacy. Self-efficacy with managing health was measured using the Managing Medications and Treatment Short Form and Self-Efficacy for Managing Chronic Conditions Short Form, which are reliable and valid in persons with stroke [65]. Each fixed-length eight-item short form was rated on a five-point scale. The scores are reported as T-scores with standard errors.

Stroke-Specific Quality of Life. The Stroke Specific Quality of Life–12 (SS-QoL) measures health-related quality of life that is specific to those living with the effects of stroke [66]. It is both valid and reliable in the stroke population [66,67]. Scores on the 12-item tool range from 1 to 5 for each item, with 1 indicating more help, being unable to execute the task, or strongly agree and 5 indicating no help needed, no trouble completing the task, or strongly disagree. The total scores are calculated out of 60, with lower scores indicating a lower quality of life.

Individualized Goal Setting and Scoring. The COPM is an outcome measure that supports the identification of performance problems [68]. The COPM was modified in this study to support the identification of occupational performance problems related to vascular risk factors. The COPM was administered during the first treatment session and last treatment session by trained study personnel. Each identified problem was rated a on 10-point scale for performance and satisfaction (lower scores indicate worse performance/satisfaction). Pre- and post-intervention performance and satisfaction scores were used to evaluate treatment effect.

## 3. Data Analysis

Study data were collected and managed using REDCap electronic data capture tools hosted at UNM. REDCap (Research Electronic Data Capture) is a secure, web-based softwared platformed designed to support data capture for research studies (Harris et al., 2019) [69]. Descriptive statistics were used to characterize the data, examine trends, and evaluate feasibility, fidelity, and acceptability.

## 4. Results

### 4.1. Participants

The participants’ demographic and clinical characteristics are detailed in Table 2. The participants were randomized to treatment or waitlist control groups to determine the feasibility of the randomization scheme. One participant discontinued participation after waitlist control baseline and post-intervention testing visits due to a problem collecting primary communication information. The sample of participants (N = 8) was well-educated, with six of the eight having attended at least some college. Four of the eight participants were non-Hispanic White. The participant’s EF scores, self-efficacy scores, and stroke-specific quality of life scores are detailed in Table 2. The participant risk scores are detailed in Table 3. Of note, six of the eight participants were classified as aphasic and were recruited from a UNM stroke registry listserv of people with a history of aphasia.

### 4.2. Feasibility

**Figure 1:**
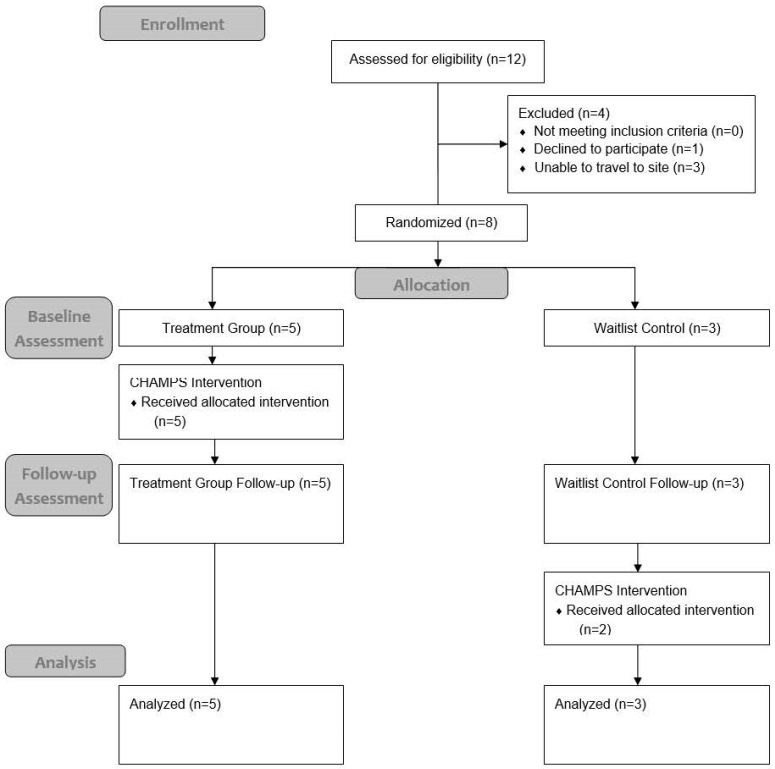
Consort Flow Chart.

Refer to Figure 1 for Consort flow chart of the participants’ engagement in the study. This case series found that among the CHAMPS trial participants, 7/8 completed all the study elements with no serious adverse events reported. The eight participants were recruited over the span of 3 months. Of the 12 people directly contacted who were interested in the study, 3 were not eligible due to travel constraints for the testing visits. Two of the enrolled participants opted for in-home assessment visits as community mobility was challenging. The recruitment rate was 66.7% and the retention rate was 87.5%, with a drop-out proportion of one drop out and seven completed entries (1:7). The study was deemed acceptable based on the retention rate. The fidelity of the delivered intervention ranged from 87.5 to 100% (m = 95.83%) for the participants and was derived from the study logs and drop-in fidelity checks. Notably, all the participants with aphasia successfully completed the intervention with minimal adaptations made by the trained study personnel (e.g., incorporating strategies for verbal communication during testing and intervention sessions). The data from our detailed study logs showed that the participants generally reported that the structured, yet flexible format of CHAMPS was motivating and manageable. Several noted that having concrete, personalized goals made the sessions feel directly relevant to their daily lives. Others appreciated the predictability of the virtual sessions, especially when paired with reminders and visual supports. For those with aphasia or executive challenges, the ability to revisit strategies weekly and practice them in real-life contexts enhanced their confidence. A few participants expressed difficulty navigating the technology early on, but reported improved ease with continued support and repetition.

### 4.3. Safety

No serious adverse events were reported during the duration of the study. Many of the participants had chronic pain related or unrelated to the stroke, which restricted the ability to fully engage in the physical activity recommendations. Nonetheless, the study personnel provided education on strategies to “move more” in pain-free positions and in smaller, more manageable amounts of time throughout the day.

### 4.4. Delivery Characteristics

The up-to-10 bi-weekly treatment sessions were deemed appropriate for all the participants. Several participants were non-smokers (N = 6) and were not diabetic or pre-diabetic (N = 5) and thus completed fewer sessions for personally irrelevant content. The average length of the baseline testing visit was 120 min, and the first session, which also included COPM delivery, was 67.5 min. The subsequent treatment visits averaged 40 min. Missed treatment times were minimal as the research personnel provided reminders of upcoming sessions the day prior and/or the morning of the scheduled visit. Accessibility issues arose and were documented, which included adding tabs to easily locate sections of the notebook and managing a smartphone for videoconferences with hemiparesis.

### 4.5. Potential Effects

The COPM was completed at the first and last treatment sessions; pre- and post- performance and satisfaction scores were collected based on each participant’s self-identified goals (Table 4). For performance, the average group change and standard deviation was 1.22 ± 0.87. For satisfaction, the average group change and standard deviation was 1.18 ± 0.83. The goals shifted for some participants (Case 3 and Case 4) as they worked through the intervention and became more aware of the limitations that were increasing their risk. For instance, Case 4 added medication management as a goal and Case 3 added goals for sleep and managing blood sugar. Importantly, not all COPM scores improved. In fact, some scores remained unchanged or decreased. These score patterns may reflect an increase in self-awareness rather than a decline in ability. As the participants gained insight into their EF impairments and health behaviors, they may have reassessed their performance or satisfaction more critically. This shift in awareness can be a positive outcome of metacognitive training. Future trials may benefit from using Goal Attainment Scaling or incorporating objective health metrics to more accurately capture functional changes alongside evolving self-perceptions.

Refer to Table 3 for average pre- and post- treatment goal performance and satisfaction ratings.

## 5. Discussion

A feasibility study is an essential first step prior to a scaled trial because it helps evaluate whether the intervention can be delivered as intended and supports the identification potential barriers to delivery. The purpose of this study was to evaluate the feasibility, acceptability, safety, fidelity, suitability, and intervention characteristics. This case series study found that among the CHAMPS trial participants, 8/9 completed all the elements and no serious adverse events were reported. Additionally, participants’ engagement with the CHAMPS intervention was strong, as evidenced by the high retention rate. The high retention rate was attributed to the close contact we kept with the participants and the open lines of communication we maintained. The preliminary findings suggest that the CHAMPS trial is safe, feasible, and acceptable among people with post-stroke EF impairments. The results of this study align with previous research showing that it is feasible and acceptable to combine metacognitive strategy training with health behavior coaching [70,71].

The flexibility of the CHAMPS intervention, which included both in-home visits and virtual sessions, was crucial in accommodating participants with mobility challenges. This is significant given that people living with the effects of stroke may have difficulty with community mobility compared to neurologically healthy adults. Even those with mild stroke that tend to experience the cognitive effects of stroke have difficulty navigating their community due to difficulty with driving and using public transportation [3]. People with chronic stroke continue to experience difficulty accessing their community due to limitations and environmental barriers [72]. Our team adjusted the protocol to include in-home visits as two participants had great difficulty traveling within the city to the baseline testing visit site. This adjustment afforded our team the opportunity to connect with participants that may be in greatest need of interventions such as that offered in the CHAMPS study. The participants appreciated the virtual nature of the intervention visits and many desired a fully remote program where testing visits could be conducted virtually as well.

A population that is often excluded from stroke rehabilitation research studies are those who experience aphasia. Research protocols often require verbal or written responses, which create a barrier to participation for those with aphasia. In fact, a recent systematic review showed that in RCT research, only 6.5% of studies explicitly included people with aphasia [73]. This is highly concerning as aphasia affects nearly one-third of people living with stroke [74]. Our study included participants with mild to moderate aphasia, as screened by the NIHSS. The participants with post-stroke aphasia in our study were able to complete the baseline and follow-up testing (N = 6) and the intervention (N = 5). The one participant with aphasia who did not complete the intervention was lost to follow-up due to an email/phone miscommunication; his engagement was not limited by his aphasia.

The CHAMPS study was not designed to test the intervention’s effects; thus, further studies will need to be conducted to determine if the intervention reduces vascular risk and increases self-efficacy. Nonetheless, our study found that people with post-stroke EF impairments who participated in the intervention experienced improvements in performance and satisfaction in performance of goals related to health self-management and vascular risk reduction. It was noteworthy that the participants became more aware of their EF limitations and self-management problems during the intervention sessions, and this may have influenced some participants’ self-ratings that decreased after the intervention compared to before the intervention. Self-awareness of EF impairments should be considered in future projects.

CHAMPS represents a significant innovation in stroke rehabilitation by combining multiple evidence-based approaches into a unified, accessible format. It goes beyond conventional telerehabilitation, which often emphasizes physical recovery or general support, by embedding cognitive strategy training directly into the structure of vascular risk reduction strategies. Unlike traditional health coaching, CHAMPS is tailored to the needs of individuals with EF impairments and recognizes that challenges with initiation, planning, and self-monitoring require consideration. Additionally, CHAMPS differs from typical occupational therapy sessions in its structured use of the CO-OP approach and digital tools to reinforce metacognitive learning and strategy generalization in everyday life.

Methodological issues and limitations were present in this study and further discussion is warranted. First, self-reporting was used in this study for pragmatic reasons but objective tracking would better support both self-awareness and testing to assess the efficacy of the intervention in future studies. Second, the sample size was small and to obtain statistically significant results, a larger sample size is necessary.

The management of vascular risk factors, such as hypertension, diabetes, smoking, and cholesterol levels, can reduce the risk of recurrent stroke that can lead to greater severity of disability post stroke. Ischemic strokes can trigger pathophysiological mechanisms that contribute to vascular dementia and recurrent stroke in and of itself and can lead to an increased prevalence of dementia (reported to be up to 30%) [19,75]. The Finnish Geriatric Intervention Study to Prevent Cognitive Impairment and Disability (FINGER) has shown improved cognitive outcomes with multidomain lifestyle interventions and both vascular and metabolic risk monitoring [76,77]. Interventions should target people at heightened risk for dementia like those with manifested vascular disease like stroke. In fact, dementia prevention interventions are more successful in high-risk persons with vascular disease before the manifestation of dementia [78]. Multidomain lifestyle interventions that integrate tailored coaching and cognitive strategies can support self-management goals for vascular risk reduction when EF impairments contribute to difficulties with behavior changes (e.g., impulsive eating and mental flexibility when one is unable to go to the gym because of a schedule change).

The CHAMPS trial, albeit feasible, had several limitations due to the relatively short timeline and limited funding that will be addressed in a future trial. First, unless the participants had access to recent labs that they were willing to share, the objective physiological metrics for HbA1c and lipid levels were self-reported. Although the effects of the intervention were not evaluated in this study, having objective data could have allowed for better characterization of the participants, more tailored AHA LE8 content, and professional recommendations if needed. Another limitation was that this study used the risk score calculation methodology developed for AHA LS7 instead of LE8 due to how the data were collected. A formal usability assessment (e.g., the System Usability Scale) and analytics on the platform were not conducted. Future trials should include standardized measures to evaluate technology accessibility and usability as people with stroke have various impairments that can influence technology use. Although the intervention materials were designed to be accessible, some challenges arose in this study. The materials will be updated in a subsequent trial to further improve accessibility. Another limitation is that our feasibility study initially employed a waitlist control design to ethically provide access to the intervention for all participants. This design however necessitated adaptations to our pre-defined go/no-go criteria. A no-go was triggered as the waitlist dropout was >20%, indicating that the waitlist period gave the perception that waiting negatively impacted the study. Therefore, we removed the waitlist control element and all the participants were enrolled in the intervention thereafter. While findings of this study are promising, their generalizability is limited by the small, relatively well-educated sample, most of whom were years post-stroke. We acknowledge that missing data limited our ability to characterize the population; however, this does provide insight on expectations for missing data in future scaled trials. These characteristics may have enhanced engagement with the virtual platform. Inclusion of participants with mild to moderate aphasia supports broader accessibility, though future trials should assess CHAMPS in more diverse populations in terms of education, stroke chronicity, and cognitive severity.

### Broad Impacts

The findings from this feasibility study meaningfully contribute to the evolving field of assistive technology and technology-based rehabilitation by demonstrating how commercially available teleconferencing platforms can be adapted for interventions and partially serve as cognitive prosthetic tools. Although CHAMPS was delivered by an occupational therapy team, future trials should strategically integrate nursing into the delivery. Our team frequently consulted with nurses during this trial, but involving nurses in protocol refinement and future trial delivery would be beneficial to the participants, particularly those with greater health-related needs. The virtual format of CHAMPS positions it for integration into community-based stroke programs, especially for patients who are not receiving services after being discharged from healthcare. With the growing number of telehealth reimbursement models and alignment with chronic care management goals, CHAMPS may offer a cost-effective way to support recurrent stroke prevention.

This work underscores the potential for scalable, technology-enabled interventions to extend cognitive rehabilitation beyond traditional clinical settings, particularly for individuals with limited access to in-person care due to geographic, mobility, or socioeconomic barriers. By integrating well-established cognitive intervention strategies within a virtual framework, this study advances the design of user-focused interventions that support neurocognitive recovery and self-management. Future trials should further integrate technology to enhance the reach and scalability of the intervention. This study was limited in its geographic reach and modifying the protocol to be fully virtual could offer opportunities for more participants to engage in the trial. In summary, these insights support the next steps for a scaled efficacy trial that strategically incorporates an interdisciplinary approach for a fully virtual protocol that meets the real-world needs of people living with the effects of stroke.

## 6. Conclusions

Adults living with the effects of stroke may have underlying EF impairments that make the adoption of health behaviors for vascular risk reduction particularly challenging. CHAMPS was developed to address this problem by virtually delivering a self-management coaching and cognitive strategy program that integrates vascular health education coaching with metacognitive training. This case series study, although small, demonstrated feasibility, acceptability, and the potential to improve self-management of vascular risk factors among individuals with post-stroke EF impairments. Beyond confirming that CHAMPS can be delivered virtually, the findings offer insight into how and why it may promote meaningful engagement, including its tailored approach to cognitive barriers and the use of technology as a cognitive prosthetic. A scaled efficacy trial is warranted to further evaluate the CHAMPS intervention.

## Figures and Tables

**Table 1:** Demographic and clinical characteristics of participants.

	Case 1	Case 2	Case 3	Case 4	Case 5	Case 6	Case 7	Case 8
Age	48	53	61	50	55	75	66	86
Sex	Female	Male	Male	Male	Male	Male	Female	Female
Race/Ethnicity	Hispanic/ White	Non-Hispanic White	Hispanic/ White	Non-Hispanic White	Hispanic/ White	Non-Hispanic White	Hispanic/White	Non-Hispanic White
Education Level	High School	High School	Some College	College Graduate	College Graduate	Some College	College Graduate	College Graduate
Marital Status	Never Married	Married	Couple	Married	Married	Married	Divorced	Widowed
Work Status	Unable to work	Retired	Retired	Employed	Retired	Unable to Work	Employed	Employed
Income Level	Unsure	35 K to 50 K	15 K to 35 K	75 K or more	75 K or more	35 K to 50 K	75 K or more	75 K or more
Time since Onset	7 years	8 years	8.5 years	4 years	8 years	1.5 years	1 year	<1 year

**Table 2:** Participant cognitive characterization, self-efficacy, and stroke-specific quality of life.

	Case 1	Case 2	Case 3	Case 4	Case 5	Case 6	Case 7	Case 8
NIHSS Total Score	7	1	5	2	2	1	0	0
OxMET Accuracy Rule Breaks	−6 15	3 6	−3 12	4 5	2 8	8 2	8 2	6 4
Menu Task Assessment Total	9	10	4	8	11	8	11	7
NIH Toolbox Fully Corrected T-Score *								
Flanker Inhibitory Control Percentile	20 5	32 4	31 5	28 3	38 16	50 38	55 69	20
Dimensional Card Sort Percentile	28 10	40 15	39 2	42 35	42 15	43 26	51 54	28
List Sorting Working Memory Percentile		48 39	36 5	47 48	32 4	17 80	42 23	
Pattern Comparison Processing Speed Percentile	36 11	33 3	39 22	41 12	47 44	38 13	47 44	43 54
Promis SE Managing Chronic Conditions T-score (SE)	50.26 (2.23)	48.18 (2.17)	58.35 (3.41)	50.26 (2.23)	58.35 (3.41)	46.27 (2.15)	50.26 (2.23)	63.85 (5.39)
Promis SE Managing Medications and Treatment T-score (SE)	45.20 (2.80)	38.30 (2.32)	60.74 (6.31)	54.95 (4.66)	60.74 (6.31)	30.44 (2.17)	49.91 (3.73)	60.74 (6.31)
SSQoL	47	39	46	31	43	37	46	57

* NIH Toolbox Raw Scores are reported instead of Fully Corrected T-Scores for Case 1 and Case 8 due to missing data. List Sorting scores were not obtained due to not having full access to the assessment within the participant’s homes. Refer to assessment section for score information for each measure used.

**Table 3:** Risk scores.

	Case 1	Case 2	Case 3	Case 4	Case 5	Case 6	Case 7	Case 8
LE8 Risk Score	7	6	8	7	11	8	11	14
mCAIDE Risk Score	5	8	6	6	2	4	2	5

Refer to assessment section for information for each measure used.

**Table 4:** Mean (±SD) of COPM performance and satisfaction scores for each case.

	Pre	Post
Individual Scores		
Case 1		
Performance	3.67 ± 2.52	5.00 ± 2.64
Satisfaction	3.67 ± 2.31	5.33 ± 3.06
Case 2 (lost to follow up)		
Performance	-	-
Satisfaction	-	-
Case 3		
Performance	5.00 ± 2.16	8.33 ± 1.53
Satisfaction	5.75 ± 1.26	9.33 ± 1.15
Case 4		
Performance	4.00 ± 0.00	3.67 ± 2.51
Satisfaction	7.00 ± 0.00	4.00 ± 1.00
Case 5		
Performance	6.00 ± 1.00	7.00 ± 1.00
Satisfaction	3.67 ± 2.31	4.67 ± 2.31
Case 6		
Performance	8.00 ± 0.00	8.00 ± 0.00
Satisfaction	8.00 ± 1.00	9.00 ± 0.00
Case 7		
Performance	6.00 ± 0.00	8.00 ± 0.00
Satisfaction	6.00 ± 0.00	9.00 ± 0.00
Case 8		
Performance	4.25 ± 2.52	5.50 ± 1.91
Satisfaction	3.50 ± 1.00	4.50 ± 1.29

## Data Availability

Data is contained within the article.
